# Improved internal control for molecular diagnosis assays

**DOI:** 10.1016/j.mex.2015.03.002

**Published:** 2015-03-12

**Authors:** T. Vinayagamoorthy, Danielle Maryanski, Dilanthi Vinayagamoorthy, Katie S.L. Hay, Jacob Yo, Mark Carter, Joseph Wiegel

**Affiliations:** MultiGEN Diagnostics LLC, 854 Pargon Way, Rock Hill, SC 29730, United States

**Keywords:** True negative control for PCR amplification and DNA sequencing coupled analysis, Multiplex PCR, Nucleic acid amplification test, Sanger sequencing, Analyte, Pb2a, IC-Code

## Abstract

The two principal determining steps in molecular diagnosis are the amplification and the identification steps. Accuracy of DNA amplification is primarily determined by the annealing sequence of the PCR primer to the analyte DNA. Accuracy for identification is determined either by the annealing region of a labelled probe for the real time PCR analysis, or the annealing of a sequencing primer for DNA sequencing analysis, that binds to the respective analyte (amplicon). Presently, housekeeping genes (Beta globin, GAPDH) are used in molecular diagnosis to verify that the PCR conditions are optimum, and are thus known as amplification controls [1], [2], [3], [4]. Although these genes have been useful as amplification controls, they lack the true definition of an internal control because the primers and annealing conditions are not identical to the analyte being assayed. This may result in a false negative report [5]. The IC-Code platform technology described here provides a true internal control where the internal control and analyte share identical PCR primers annealing sequences for the amplification step and identical sequencing primer annealing sequence for the identification step.

• The analyte and internal control have the same PCR and sequencing annealing sequences.
• This method assures for little or no false negatives and false positives due to the method’s design of using identical annealing conditions for the internal control and analyte, and by using DNA sequencing analysis for the identification step of the analyte, respectively.
• This method also allows for a set lower limit of detection to be used by varying the amount of internal control used in the assay.

## Method

### Primer design

**Fig. 1:**
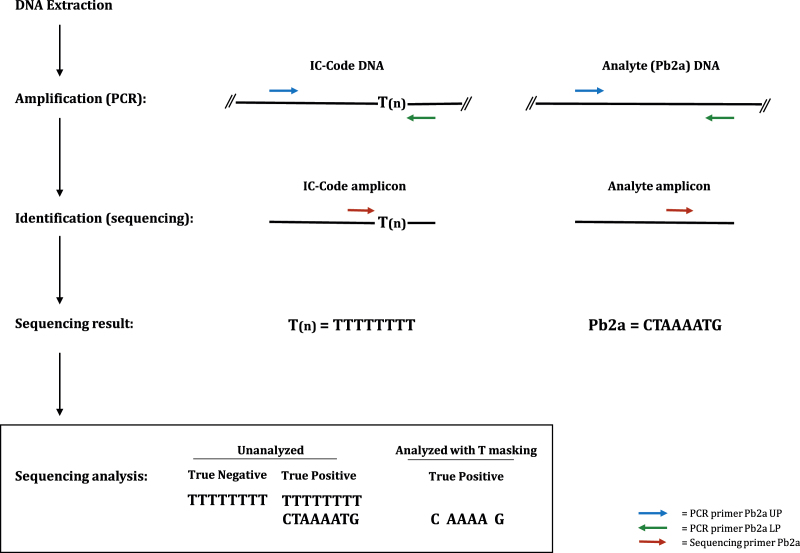
Schematic flowchart and expected data for the application of IC-Code™ for molecular diagnostic assays.

IC-Code™ is a DNA sequencing platform technology to develop nucleic acid amplification test (NAAT) with true internal control. The scientific concept of IC-Code™ is based on using a synthetic DNA construct that has the same PCR and sequencing primer annealing sequences as that on the analyte (Fig. 1). The analyte and synthetic DNA construct will have identical sequencing primer annealing sites located approximately 25 bp internal of either the downstream or forward primer, with sequencing being targeted toward that primer. Therefore, the sequence generated will represent only that of 25 bp region. To distinguish the internal control amplicon from the analyte amplicon, the nucleotide sequence between the 3′end of the PCR primer and 3′ end of the sequencing primer will carry a homo-nucleotide sequence (dTTP in this report) to tag the internal control. PCR primers used were Pb2a UP: ATGATATAAACCACCCAATTTGTCTGCCAGTTTCTCCTTG and Pb2a LP: TCAATCTATAGCGCATTAGAAAATAATGGCAATATTAACGCACCTC. Sequencing primer used was Pb2a SP: TTATGCAAACTTAATTGGCAAATCCGGTAC.

## Sequencing analysis

The synthetic DNA is either added externally before the sample is processed or will be in the sample collection tube itself. In the absence of the analyte (true negative), the PCR primers will generate a single amplicon using the synthetic DNA of the IC-Code™ as its template (Fig. 1). During sequencing of this amplicon, the sequencing primer will bind to its annealing site on the synthetic DNA internal control and generate a unique poly-T sequence. However, in the presence of an analyte (true positive), two amplicons will be generated with one from the analyte and the other from the synthetic internal control, and both will be sequenced simultaneously (Fig. 1). Therefore, the electropherogram will show mixed bases from both amplicons. In order to visualize the analyte nucleotide sequence, T signals are masked from the sequences generated by the two amplicons using a feature in the analysis software (Genetic analyzer, Life technologies, USA). When all the T-peaks generated from the internal control are masked then the sequences from the other three nucleotides remain, which is sufficient to identify the analyte [6].

## Recommended equipment

• Capillary electrophoresis Genetic analyzer 3130 series [7].
• Thermocyclers Gene amp 9700 [8].
• Thermocyclers Gene amp 2720 [8].

Comparable products can be used for all three equipment.

## Sample preparation

1. A region encompassing part of the methicillin resistant gene Pb2a was amplified using *Staphylococcus aureus* genomic DNA obtained from American Type Culture Collection (ATCC) and Pb2a specific primers (MultiGEN Diagnostics LLC, USA).
2. Each reaction mixture included 25 μl of Master Mix 2X buffer (Multiplex PCR Plus Kit Qiagen, USA), 1 μl each of forward and reverse primers at 10 pmol/μl, 1 μl of target template at 0.4 ng/μl, 1 ul of IC-Code template at 0.28 ng/μl, and 21 μl of water. Based on the estimated lower limit of detection (LLOD), the amount of the IC template added can be varied so that the signals generated from the IC and the target does not suppress each other.
3. PCR conditions consisted of an initial denaturation at 95 °C for 5 min; 35 cycles of 95 °C for 30 s, 65 °C for 90 s, and 72 °C for 30 s; and a final extension at 68 °C for 10 min.
4. After PCR, 5 units of uracil-DNA glycosylase were added to the reaction and incubated for 60 min at 37° C and deactivated at 96 °C for 3 min.
5. The amplicons were cleaned using Ampure (Beckman Agencourt, USA).
6. Purified amplicons (2.5 μl) were sequenced in a 10 μl reaction volume using 1 μl Pb2a sequencing primer (MultiGEN Diagnostics Inc., USA) at 5 pmol/μl, 1 μl of ABI PRISM Big Dye Terminator Ready Reaction Mix version 1.1 (Life Technologies, USA), 1.5 μl BigDye Terminator 5X Sequencing Buffer version 1.1, 3.1 (Life Technologies, USA), and 4 μl water. The sequencing conditions consisted of 25 cycles of 96 C for 10 s, 52.5 C for 10 s and 60 C for 2.5 min. Unincorporated dye terminators were removed using CleanSEQ (Beckman Agencourt USA). Samples were analyzed by capillary electrophoresis using the ABI PRISM Genetic Analyzer 3130.1

## Method validation

**Fig. 2:**
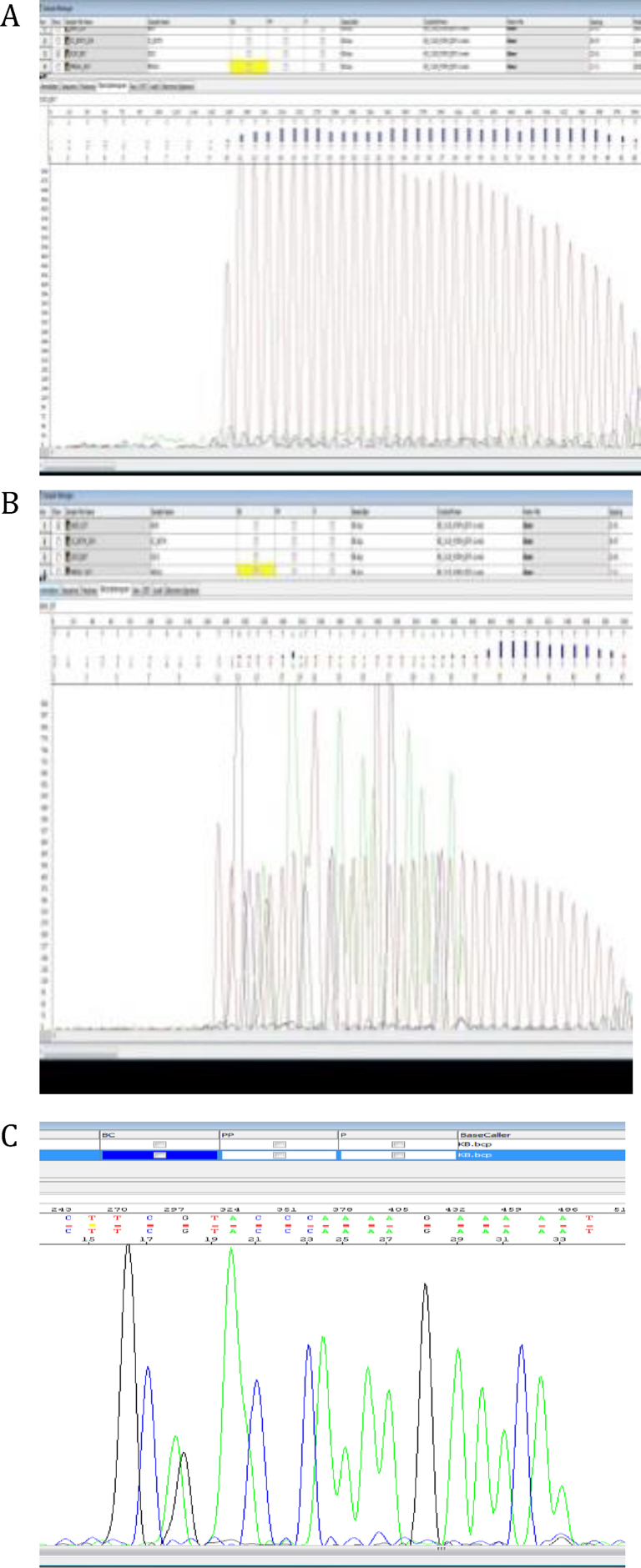
Sequencing analysis results for IC-Code™ (internal control) and Pb2a (analyte). (A) Electropherogram for IC-Code™ only – true negative. (B) Electropherogram for IC-Code™ and Pb2a mixed – true positive. (C) Post-analysis of true negative with T-peaks (red) removed, leaving only A (green), C (blue), and G (black) peaks for base calling. (For interpretation of the references to color in this figure legend, the reader is referred to the web version of this article.)

To test the efficacy of the IC-Code™ to act as a true internal control, the IC-Code™ was tested by itself as a “true negative”. This negative result (IC-Code only) is shown in Fig. 2A with a clean poly-T profile detected, as expected. In contrast, when the Pb2a template was added to the reaction the electropherogram profile showed mixed based calls, indicating sequences from two different priming sites, or from two different amplicons as in this experimental approach (Fig. 2B). However, when the same ABI file was re-analyzed with the T signal channel switched off, only the sequences of the remaining three dideoxynucleotides from the anaylate were seen (Fig 2c). The partial base call from the re-analyzed file matched the expected base call for Pb2a (Table 1).

## Additional information

False negative reports could be devastating to patient care and should be prevented for optimum patient care. Therefore, one of the most crucial goals in molecular diagnostics is to avoid a false negative. Although there are number of reasons for a false negative, two main reasons are: polymorphism at the primer binding sites of the analyte and detecting close to the LLOD so that the signal from the analyte can be distinguished from the noise. By varying the amount of synthetic DNA introduced as IC template, a desired LLOD can be established with confirmation that there are no false negatives at the set LLOD. These features should significantly reduce the incidence of false negative reports. This study provides proof-of-principle data for IC-Code™ that can be used in molecular diagnostics, including next generation sequencing. Future experiments will test the IC-Code™ platform with clinical samples to measure its effectiveness in lowering the percentage of false negative reports against other commonly used platforms. Finally, there are many applications for the IC-Code™ platform technology and some of the most important ones are listed below.

### Methicillin resistant *S. aureus* (MRSA)

MRSA infection is becoming the lead pathogen among hospital borne infection [9], [10]. Presently, MRSA is detected indirectly by identifying the staphylococcus chromosome cassette (SCC) and not directly by the more specific region encoding the methicillin resistant gene, Pb2a. This may lead to false positive results. Hence, the IC-Code™, with direct amplification and sequencing of the Pb2a gene would significantly reduce the percentage of false positive results (see Fig 2).

### Blood transfusion

In the United States, more than 15.7 million blood donations are collected annually from 9.2 million people to meet the daily requirements of 41,000 blood product transfusions [11]. These are usually administered as various components, such as packed red cells, plasma, and platelets. Patients who require general surgery, resuscitation from major trauma, transplants, and chemotherapy, are the major recipients for blood product transfusions. Hence, ensuring the safety of these blood products is vital. Therefore, screening is used to test for the presence of various transfusion-transmitted pathogens (Hepatitis B, Hepatitis C, HIV, and West Nile Virus). These screens require the need of a true internal control to prevent the use of contaminated blood products due to a false negative result.

In addition to screening stored blood products, blood samples are screened for bacterial meningitis (Group B *Streptococcus, Escherichia coli, Listeria monocytogenes Streptococcus pneumoniae, Neisseria meningitidis, Haemophilus influenzae* type B) in new born and infants, and for viral meningitis (Coxsackievirus A, B, Echo virus, Enterovirus D68). These are serious and life threatening conditions that need accurate detection without false negative reports.

### Septicemia

Bodily fluids, including blood, are generally considered pathogens free. But pathogens do invade bodily fluids and may cause septicemia. Most cases of septicemia are through infection of lungs, abdomen and urinary tracts caused by bacteria (*Staphylococcus pyogenes, E. coli, Pseudomonas aeruginosa, Klebsiella*) and fungi (*Candida* spp.). Early positive identification of an infection is critical for treatment and isolation to prevent the spread of infection. This may be the outcome with a false negative report. But with a true internal control, these false negative reports can be avoided

### Bio processing

Manufacturing processes involving cells may consist of upstream processes that include animal products (fetal bovine serum). Some of the microbes tested in these animal products include *Bacillus cereus, Mycoplasma, Pseudomonas*, and *E. coli*. After product development, the next step is purification of the product for desired quality through downstream processes that may include filtration. Validation and quality control of the filtration process requires a foolproof testing system where no false negatives can be tolerated. Using IC-Code™ will reduce false negative reports and further, if a positive contaminant is detected, the identity could be confirmed without any delay so that immediate action could be implemented.

## Figures and Tables

**Table 1:** Base calls for analyzed Pb2a. Upper row is complete primary sequence for Pb2a. Lower row is analyzed data with T-peaks masked.

Pb2a sequence	T	G	C	A	G	A	A	C	T	A	A	A	A	T	G	A	A	A	C	A	A
Analyzed data		G	C	A	G	A	A	C		A	A	A	A		G	A	A	A	C	A	A
